# Enhanced Neuroprotective Effects of Coadministration of Tetrandrine with Glutathione in Preclinical Model of Parkinson's Disease

**DOI:** 10.1155/2015/931058

**Published:** 2015-11-18

**Authors:** Xiang-Yun Li, Guang-Hai Mei, Qiang Dong, Yu Zhang, Zhuang-Li Guo, Jing-Jing Su, Yu-Ping Tang, Xue-Hong Jin, Hou-Guang Zhou, Yan-Yan Huang

**Affiliations:** ^1^Department of Geriatrics Neurology, Huashan Hospital, Fudan University, Shanghai 200040, China; ^2^Department of Neurosurgery, Huashan Hospital, Fudan University, Shanghai 200040, China; ^3^Department of Neurology, Huashan Hospital, Fudan University, Shanghai 200040, China; ^4^Department of Emergency Neurology, The Affiliated Hospital of Medical College Qingdao University, Qingdao 266021, China; ^5^Department of Neurology, Shanghai 9th People's Hospital Affiliated to Shanghai Jiao Tong University School of Medicine, Shanghai 200011, China; ^6^Department of Neurology, Suzhou Municipal Hospital Affiliated to Nanjing Medicine University, Suzhou 215001, China

## Abstract

*Aim*. In this study we examined the influence of tetrandrine (Tet) on the neuroprotective effects of glutathione (GSH) in the 6-hydroxydopamine- (6-OHDA-) lesioned rat model of Parkinson's disease (PD).* Methods*. Levels in the redox system, dopamine (DA) metabolism, dopaminergic neuronal survival, and apoptosis of the substantia nigra (SN) and striatum, as well as the rotational behavior of animals were examined after a 50-day administration of GSH + Tet (or GSH) and/or L-3,4-dihydroxyphenylalanine (L-dopa) to PD rats. Ethics Committee of Huashan Hospital, Fudan University approved the protocol (number SYXK2009-0082).* Results*. Administration of GSH or Tet alone did not show any significant effects on the factors evaluated in the PD rats. However, in the GSH + Tet group, we observed markedly decreased oxidative damage, inhibition of DA metabolism and enhanced DA synthesis, increased tyrosine hydroxylase- (TH-) immunopositive neuronal survival, and delayed apoptosis of dopaminergic neurons in the SN. Animal rotational behavior was improved in the GSH + Tet group. Additionally, coadministration of GSH + Tet appeared to offset the possible oxidative neurotoxicity induced by L-dopa.* Conclusion*. In this study, we demonstrated that tetrandrine allowed occurrence of the neuroprotective effect of glutathione probably due to inhibition of P-glycoprotein on 6-hydroxydopamine-lesioned rat models of Parkinson's disease, including rats undergoing long-term L-dopa treatment.

## 1. Introduction

Parkinson's disease (PD), characterized by the progressive loss of dopaminergic neurons mainly in the substantia nigra (SN) [1], is a common neurodegenerative disease. Although the etiopathogenesis of PD remains unclear, oxidative stress and mitochondrial dysfunction are now recognized as key events in dopaminergic neuronal loss [2, 3]. Furthermore, dopamine replacement therapy with L-3,4-dihydroxyphenylalanine (L-dopa), a first-line and the most effective drug targeting motor symptoms of PD, might induce adverse effects via oxidative stress [4].

Due to endogenous dopamine (DA), iron, and neuromelanin, the SN is especially vulnerable to oxidative stress. However, glutathione (GSH) deficiency in SN is another important factor for oxidative stress [5]. GSH is the major antioxidant and redox modulator in the brain. Decreased level of GSH in SN had become a great concern long before. It was found to be the earliest known abnormal change in nigrostriatal degeneration of PD patients, followed by decreased mitochondrial complex I (CI) activity and DA levels [6, 7]. GSH levels and DA content are not selectively damaged in either SN, other brain areas of PD patients, or other diseases involving dopaminergic pathway [8, 9]. Thus, restoration of GSH level in SN, aiming at protecting DA neurons from oxidative stress injuries, might be a potential alternative treatment for PD.

Despite all this, GSH has not yet been used in central nervous system (CNS) diseases, including Parkinson's disease. One of the most important reasons is that the blood-brain barrier (BBB) penetration capacity of GSH is very limited. Many researches exploring new techniques to replenish brain GSH levels have been conducted [9]. To date, few available approaches have been approved for clinical studies due to various limiting factors. Delivery of GSH across BBB is the major problem. As it is, ATP-binding cassette (ABC) transporters are important components of the BBB.

ATP-binding cassette (ABC) transporters, highly expressed on the brain capillary endothelial cells, are a class of ATP-driven substance efflux pumps. Of all ABC transporters, the function of P-glycoprotein (P-gp, ABCB1, and MDR1) is the most characterized. It has been proved that there is a close relationship between P-gp and numbers of prescribed drugs (polyspecificity) [10]. For the increased ratios of brain-to-plasma drugs concentration, P-gp knockout mice have been used extensively in scientific researches [11, 12]. Because GSH is also a substrate of P-gp [5], inhibition/deficiency of P-gp might lead to enhanced GSH delivery to the CNS.

Tetrandrine (Tet), a benzylisoquinoline alkaloid isolated from the Chinese herb Fen Fang Ji, is a potent and reversible inhibitor of P-gp. Reportedly, Tet can sensitize cells with multidrug resistance (MDR) to various anticancer drugs. The reversal of MDR by Tet was associated with the inhibition of Tet to the expression and transport function of P-gp, which enhanced the intracellular accumulation of anticancer drugs [13, 14]. In a word, these studies focused on the point that P-gp-mediated MDR in cancers might be reversed by Tet. However, until now there have been no studies on Tet involving neurodegenerative diseases.

The focus of this study was to determine whether coadministration with Tet, a potent inhibitor of P-gp, could restore the neuroprotective effects of GSH on the nigrostriatal system in 6-hydroxydopamine- (6-OHDA-) lesioned rat models of PD, as well as in PD rats undergoing long-term L-dopa treatment.

## 2. Materials and Methods

### 2.1. Materials

Apomorphine (APO), 6-hydroxydopamine (6-OHDA), reduced GSH, L-dopa, Tet, DA, and homovanillic acid (HVA) standards were obtained from Sigma-Aldrich (St. Louis, MO, USA). O-Phthalaldehyde (OPA), *β*-mercaptoethanol (2-MCE), glutamate, and aspartate standards were from Fluka (Switzerland). The rabbit anti-tyrosine hydroxylase (TH) antibody was purchased from Chemicon International (Temecula, CA, USA). The Streptavidin-Biotin Complex (SABC) Immunohistochemical Staining Kit was purchased from Imgenex (San Diego, CA, USA). The Reactive Oxygen Species (ROS) Detection Kit, Malondialdehyde (MDA) Detection Kit, GSH Colorimetric Detection Kit, and Monoamine Oxidase-B (MAO-B) Detection Assay Kit were obtained from BioVision, Inc. (Milpitas, CA, USA). Complex I Enzyme Activity Microplate Assay Kit and Mitochondria Isolation Kit were purchased from MitoSciences (Eugene, OR, USA).

### 2.2. Animals

Shanghai SLAC Laboratory Animal Co. Ltd. (Shanghai, China) provided us with the experimental animals we needed, 128 adult male Sprague-Dawley rats of 200–250 g. Ethics Committee of Huashan Hospital, Fudan University, approved all protocol of this study with number SYXK2009-0082.

### 2.3. Preparation of 6-OHDA-Induced Lesion Rat Model of PD

114 rats were anesthetized with 3.5% chloral hydrate (10 mL/kg, i.p.) and mounted on a stereotaxic apparatus (David Kopf Instruments, Tujunga, CA). According to a rat brain atlas book named* The Rat Brain in Stereotaxic Coordinates*, rats were lesioned with an injection of 6-OHDA (8 *μ*g in 4 *μ*L of 0.2% ascorbic acid in saline) into the left substantia nigra pars compacta (SNc, anteroposterior, AP: −5.0 mm, lateral, ML: +1.7 mm, dorsoventral, and DV: −7.6 mm relative to bregma) and into the ventral tegmental area (VTA) (AP: −4.6 mm, ML: +0.9 mm, and DV: −7.5 mm) at a rate of 1 *μ*L/min, as described previously with slight modification [15]. To confirm PD-like DA denervation, rotational behavior induced by i.p. apomorphine administration (APO, 0.5 mg/kg) was tested 4 weeks after surgery.

### 2.4. Animal Grouping and Drug Administration

114 rats were lesioned with stereotaxic injection of 6-OHDA into the left SNC and VTA to make preclinical animal model of Parkinson's Disease. After apomorphine test, 84 rats which were screened out were considered as valid models of Parkinson's disease. All the PD animals were randomly divided into six groups, with 14 animals in each group: (1) model group (normal saline, 10 mL/kg/day), (2) Tet group (15 mg/kg/day Tet), (3) GSH group (300 mg/kg/day GSH), (4) GSH + Tet group (300 mg/kg/day GSH + 15 mg/kg/day Tet), (5) L-dopa group (25 mg/kg/day L-dopa), and (6) GSH + L-dopa + Tet group (300 mg/kg/day GSH + 25 mg/kg/day L-dopa + 15 mg/kg/day Tet). We determined the dose of glutathione of the study through a pretest and related literature [16]. Animals of each group were administered the corresponding i.p. injection for 50 days.

The sham group consisted of 14 normal rats treated with normal saline 10 mL/kg/day. Three days after the end of drug administration, the rotational test was performed for the second time. Several indicators were examined after the rotational tests were performed. Among each group, six rats were used for High Performance Liquid Chromatography (HPLC), six rats for Reverse Transcriptase Polymerase Chain Reaction (RT-PCR), and two rats for Immunohistochemistry- (IHC-) TH and Immunoelectron Microscopy (IEM).

### 2.5. Determination of DA, HVA, and MAO-B Levels

After rotational test of the second time, animals were sacrificed and the caudate nucleus in striatal tissue of the lesion sides was carefully dissected according to the Rat Brain Atlas. Perchlorate (0.4 M, 1 mL/0.1 g brain wet weight) was added to remove protein. Then the brain tissue was homogenized in phosphate buffer (10 mM, pH 7.0) and centrifuged at 13000 r/min for 20 min at 4°C. The supernatant was recentrifuged for another 10 min at the same condition. The final supernatant was assayed for DA and homovanillic acid (HVA) using High Performance Liquid Chromatography (HPLC) with electrochemical detection as described previously with slight modification [17]. The mobile phase consisted of sodium acetate buffer (pH 4.5) including 10 g citric acid, 50 mg EDTA-Na, 50 g disodium citrate, and 50 mg OSA-methanol (90 : 10, v/v) dissolved in 1,000 mL double-distilled water with a flow rate of 1 mL/min. The contents of monoamine oxidase B (MAO-B) in the striatum of the lesion sides were determined using the MAO-B Detection Assay Kit, and the test was performed according to the manufacturer's instructions. There were six rats in each group (*N* = 6).

### 2.6. Reverse Transcriptase Polymerase Chain Reaction (RT-PCR)

Total RNA was extracted from the SN of the lesion sides using TRIzol reagent (Invitrogen Inc., Carlsbad, CA, USA). First strand cDNAs were synthesized with M-MLV reverse transcriptase (Fermentas, Lithuania). Polymerase Chain Reaction (PCR) was performed with tyrosine hydroxylase- (TH-) specific primers (sense, 5′-GTCCGCCCGTGATTTTCTGG-3′; antisense, 5′-AGGAGCGCTGGATGGTGTGAG-3′). The conditions for amplification were as follows: 4 min at 94°C for the initial denaturation, 30 cycles of 30 s at 94°C to denature, 30 s at 59.6°C to anneal, and 60 s at 72°C for extension, followed by a 5-minute final extension. The GAPDH gene (sense, 5′-ACCACAGTCCATGCCATCAC-3′; antisense, 5′-TCCACCACCCTGTGCTGTA-3′) was used as an internal control for gene expression. There were six rats in each group (*N* = 6).

### 2.7. Immunohistochemistry- (IHC-) TH

Coronal midbrain sections 15 *μ*m in thickness were obtained from formalin-fixed and sucrose-dehydrated brain tissues for immunohistochemical (IHC) staining. Brain sections were first rinsed in pure methanol and 3% H_2_O_2_ for 30 min at room temperature to inhibit endogenous peroxidase. The sections were incubated overnight with a rabbit anti-TH antibody (1 : 600 dilution) in phosphate buffer saline (PBS) containing 0.3% Triton X-100 and normal goat serum. Additionally, PBS was used instead of the primary antibody as a negative control. The sections were then incubated with a biotinylated anti-rabbit IgG (1 : 250 dilution) for 20 min, followed by incubation in asymptotic boundary conditions (ABC) solution for 20 min at room temperature. After each incubation step, sections were washed three times with PBS and were visualized using a Diaminobenzidine (DAB) kit. For quantification of the TH-immunopositive neurons, 20 brain slices from each group were analyzed. There were two rats in each group (*N* = 2).

### 2.8. Measurement of GSH, ROS, MDA, and Mitochondrial CI

Levels of ROS and MDA in the striatum and GSH in the SN of the lesion sides were determined using the ROS Detection Kit, MDA Detection Kit, and GSH Colorimetric Detection Kit, respectively, according to the manufacturer's instructions. Regarding CI activity, mitochondria of the SN were isolated and assayed as described previously [18]. There were six rats in each group (*N* = 6).

### 2.9. Measurement of Glutamate and Aspartate in the Striatum

Glutamate and aspartate were determined using the HPLC-fluorescence detection method as described by Yang et al. [19] with modifications. The chromatographic column used was a Diamonsil C18 reverse-phase column (4.6 mm × 200 mm, 5 *μ*m). The mobile phase used was potassium dihydrogen phosphate (0.1 M, pH 5.4) : methanol : acetonitrile (v/v) = 62 : 28 : 10, which was pumped at a rate of 1.0 mL/min without recycling. The excitation wavelength (Ex) of fluorescence detection was 357 nm and the emission wavelength (Em) was 455 nm. The entire duration of the chromatographic procedure was 20 min. There were six rats in each group (*N* = 6).

### 2.10. Immunoelectron Microscopy (IEM)

Observation of the coronal midbrain sections, removal of endogenous peroxidase, fixing of specimens, dehydration, and exposure to the primary and secondary TH-antibodies were performed in the same manner as described for IHC. Afterwards, specimens were postfixed with 4% paraformaldehyde and 0.05% glutaraldehyde, followed by embedding in EPON-812 and cutting with an ultramicrotome. Stained with uranyl acetate and lead citrate, the sections were then visualized in a Philips CM120 transmission electron microscope. There were three rats in each group (*N* = 2).

### 2.11. Statistical Analysis

The data before and after drug intervention within one group were analyzed using the paired *t*-test. To compare each experimental index among different groups, one-way analysis of variance (one-way ANOVA) and post hoc Scheffe's test were performed. All the analyses were conducted by blinded observers. All data were expressed as mean ± standard deviation (SD). A probability level of 0.05 was considered statistically significant.

## 3. Results

### 3.1. Difference in APO-Induced Rotational Behavior

**Figure 1:**
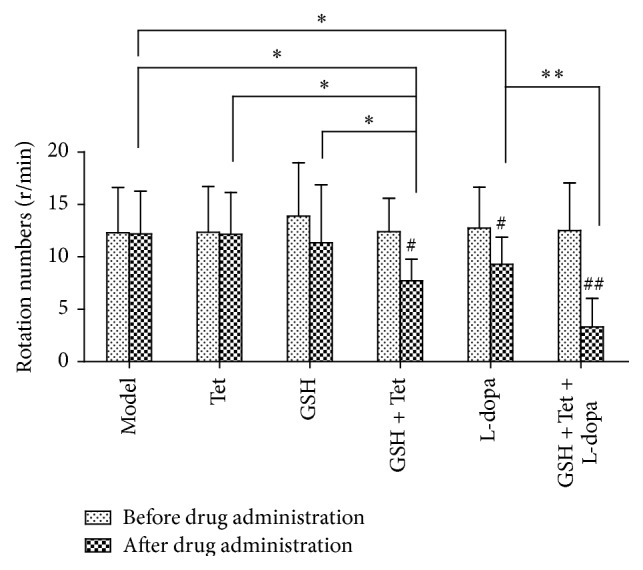
Changes in apomorphine- (APO-) induced rotational behavior of Parkinson's disease (PD) rats after drug treatment. The number of contralateral rotational turns in 30 min after intraperitoneal (i.p.) APO administration was recorded. Each bar represents mean ± standard deviation (SD) for 14 animals per group. ^#^ *p* < 0.05, ^##^ *p* < 0.01 comparison within each group before and after drug treatment. ^*∗*^ *p* < 0.05, ^*∗∗*^ *p* < 0.01 comparison among different groups after drug treatment.

Four weeks after surgery, rotational tests were performed and approximately 74% of rats were determined as successful rat models of PD. After drug administration, rotational tests were performed on the PD rats for the second time. Rotational behavior did not occur in the sham group before or after drug treatments. As shown in Figure 1, no significant differences were observed in the number of rotations after treatment in the model, Tet, and GSH groups compared with before treatment. However, in the GSH + Tet, L-dopa, and GSH + Tet + L-dopa groups, the number of rotations decreased markedly after treatment as compared with before treatment (*p* < 0.05, *p* < 0.05, and *p* < 0.01, resp.). Furthermore, the number of rotations in the GSH + Tet group after treatment decreased significantly as compared with the model, Tet, and GSH groups (*p* < 0.05 for all groups). Compared with the model group, the number of rotations after treatment decreased significantly in the L-dopa group (*p* < 0.05) and was further decreased in the GSH + Tet + L-dopa group (*p* < 0.01, compared with the L-dopa group). Additionally, several rats in the GSH + Tet + L-dopa group did not spin within 30 min after injection of APO.

### 3.2. Levels of DA, HVA, and MAO-B in the Striatum of 6-OHDA-Lesioned Sides

**Figure 2:**
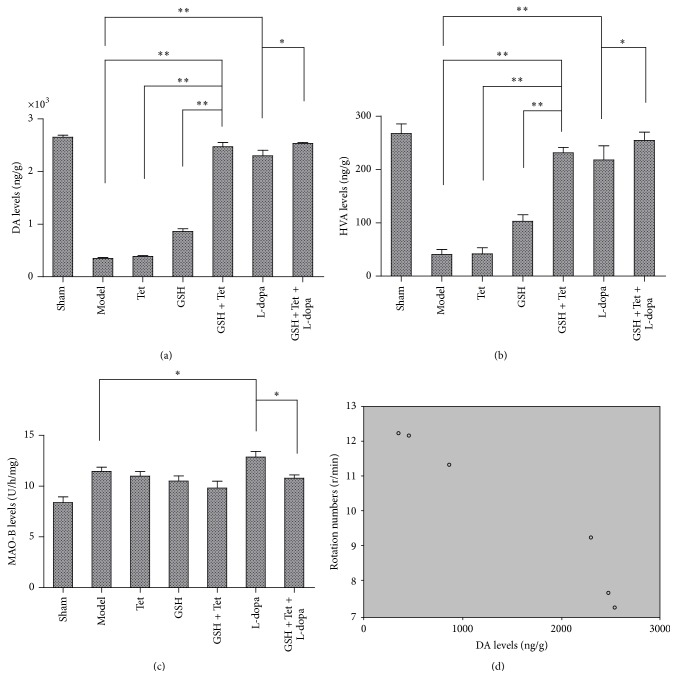
Effects on the concentration of dopamine (DA) (a), homovanillic acid (HVA) (b), and monoamine oxidase B (MAO-B) (c) in the striatum of lesion sides and the correlation between DA levels and rotational behavior induced by APO (d). Each bar represents mean ± standard deviation (SD) for six animals per group. ^*∗*^ *p* < 0.05, ^*∗∗*^ *p* < 0.01 comparison among different groups after drug treatment.

As shown in Figures 2(a) and 2(b), the levels of DA and HVA in the model group were the lowest. In the GSH + Tet group, the DA and HVA levels increased significantly compared with the model, Tet, and GSH groups (*p* < 0.01 for all groups). Additionally, the DA and HVA levels in the L-dopa group increased significantly compared with the model group as well (*p* < 0.01 for both levels). However, the GSH + Tet + L-dopa group had the highest DA and HVA levels of all groups, which increased to approximately 110.0% (*p* < 0.05) and 116.9% (*p* < 0.05), respectively, when compared with the L-dopa group. As shown in Figure 2(c), the MAO-B level was the highest in the L-dopa group, 12.4% higher than in the model group (*p* < 0.05), which was eventually offset in the GSH + Tet + L-dopa group (*p* < 0.05, compared with the L-dopa group). Additionally, correlation analysis between rotational behavior and DA levels in the striatum of lesion sides showed a strong negative linear correlation (*r* = −0.965, *p* < 0.01, Figure 2(d)).

### 3.3. Changes in TH Gene Expression in the SN of the 6-OHDA-Lesioned Sides

**Figure 3:**
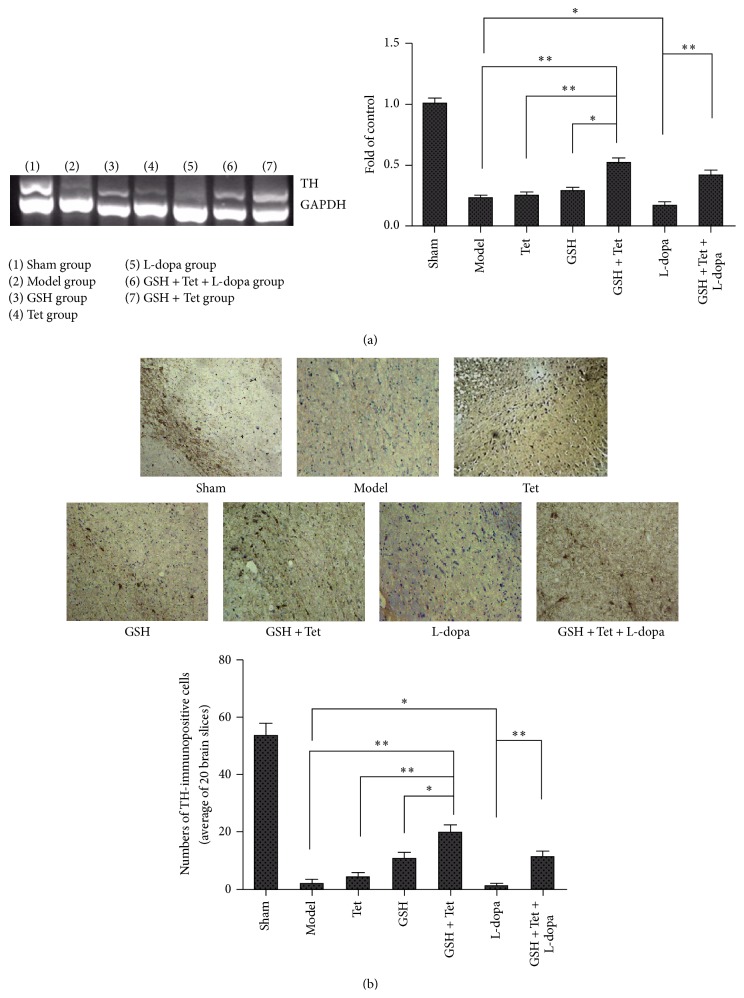
Effects on the tyrosine hydroxylase (TH) gene expression (a) and TH-positive neurons in the striatum of lesion sides (b). The expression of TH mRNA was examined by Reverse Transcriptase Polymerase Chain Reaction (RT-PCR). GAPDH served as an internal control. TH DNA quantification was obtained by densitometric analysis of the DNA band area. The TH-immunopositive neuronal cell survival was examined using immunohistochemical staining. Statistical histogram of the number of surviving TH-positive neurons represents the average of 20 brain slices from each group. Each bar represents mean ± standard deviation (SD) for six animals per group. ^*∗*^ *p* < 0.05, ^*∗∗*^ *p* < 0.01, comparison among different groups after drug treatment.

As shown in Figure 3(a), the expression of the TH gene in the GSH + Tet group increased significantly when compared with the model, Tet, and GSH groups (*p* < 0.01, *p* < 0.01, and *p* < 0.05, resp.). TH gene expression in the L-dopa group was slightly decreased compared with the model group, although not significantly (*p* < 0.05). However, in the GSH + Tet + L-dopa group, the decreased TH gene expression was reversed, resulting in an approximately 2.9-fold increase compared with the L-dopa group (*p* < 0.01).

### 3.4. TH-Immunopositive Neuronal Cell Survival in the SN of the Lesion Sides

TH-IHC was performed to determine TH-immunopositive neuronal survival and 20 brain slices from each group were analyzed. The negative control results for avoiding false positives are not shown. As shown in Figure 3(b), while many TH-immunopositive fibers and neurons were observed in the sham group, the model group had significantly fewer TH-immunopositive neurons (3.7% of the sham group, *p* < 0.001). Consistent with the RT-PCR results, TH-immunopositive neurons in the GSH + Tet group increased significantly as compared with the model, Tet, and GSH groups (*p* < 0.01, *p* < 0.01, and *p* < 0.05, resp.). The number of TH-immunopositive neurons in the L-dopa group was slightly decreased compared with the model group (*p* < 0.05). Accordingly, this reduction was effectively prevented in the GSH + Tet + L-dopa group compared with the L-dopa group (*p* < 0.01).

### 3.5. ROS and MDA Levels in the Striatum and GSH Levels and CI Activity in the SN of Lesion Sides

**Figure 4:**
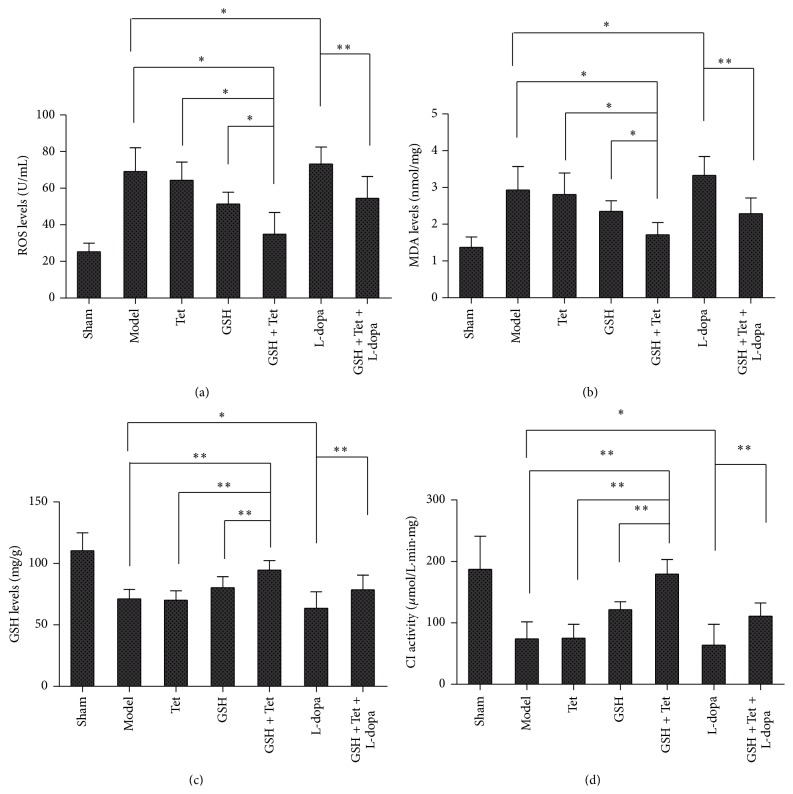
Changes in Reactive Oxygen Species (ROS) (a) and malondialdehyde (MDA) (b) in the striatum, as well as glutathione (GSH) (c) and Complex I (CI) activity (d) in the substantia nigra (SN). Bars represent mean ± standard deviation (SD) for six animals per group. ^*∗*^ *p* < 0.05, ^*∗∗*^ *p* < 0.01 comparison among different groups after drug treatment.

As shown in Figures 4(a) and 4(b), except for the sham group, the lowest levels of ROS and MDA were in the GSH + Tet group, and both levels decreased more than those in the model, Tet, and GSH groups (*p* < 0.05 for all groups). Additionally, ROS and MDA levels in the L-dopa group were the highest, which was slightly increased compared with the model group (*p* < 0.05 for both levels). In the GSH + Tet + L-dopa group, however, the ROS and MDA levels decreased markedly to approximately 74.5% and 68.8%, respectively, when compared with the L-dopa group (*p* < 0.01 for both levels). Nevertheless, the GSH levels and CI activity in each group showed an opposite pattern. As shown in Figures 4(c) and 4(d), both GSH and CI in the GSH + Tet group increased significantly compared with the model, Tet, and GSH groups (*p* < 0.01 for each group). When compared with the model group, GSH levels and CI activity in the L-dopa group decreased to approximately 89.2% and 86.1%, respectively (*p* < 0.05 for both). However, in the GSH + Tet + L-dopa group, GSH levels and CI activity significantly improved, increasing to approximately 123.6% and 174.3%, respectively, compared with the L-dopa group (*p* < 0.01 for both).

### 3.6. Glutamate and Aspartate Levels in the Striatum of the Lesion Sides

**Figure 5:**
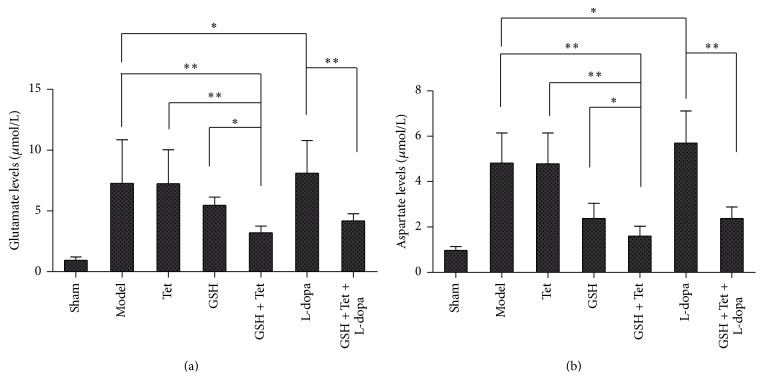
Changes in glutamate (a) and aspartate (b) in the striatum of the lesion sides after corresponding drug administration. Bars represent mean ± standard deviation (SD) for six animals per group. ^*∗*^ *p* < 0.05, ^*∗∗*^ *p* < 0.01 comparison among different groups after drug treatment.

As shown in Figures 5(a) and 5(b), glutamate and aspartate levels within each group changed consistently. Both glutamate and aspartate levels in the GSH + Tet group decreased significantly compared with the model, Tet, and GSH groups (*p* < 0.01, *p* < 0.01, and *p* < 0.05, resp.). In the L-dopa group, glutamate and aspartate levels were slightly increased compared with the model group (*p* < 0.05 for both levels) and reduced significantly to approximately 51.6% and 41.8%, respectively, in the GSH + Tet + L-dopa group when compared with the L-dopa group (*p* < 0.01 for both levels).

### 3.7. Ultrastructural Changes of Apoptotic Dopaminergic Neurons

**Figure 6:**
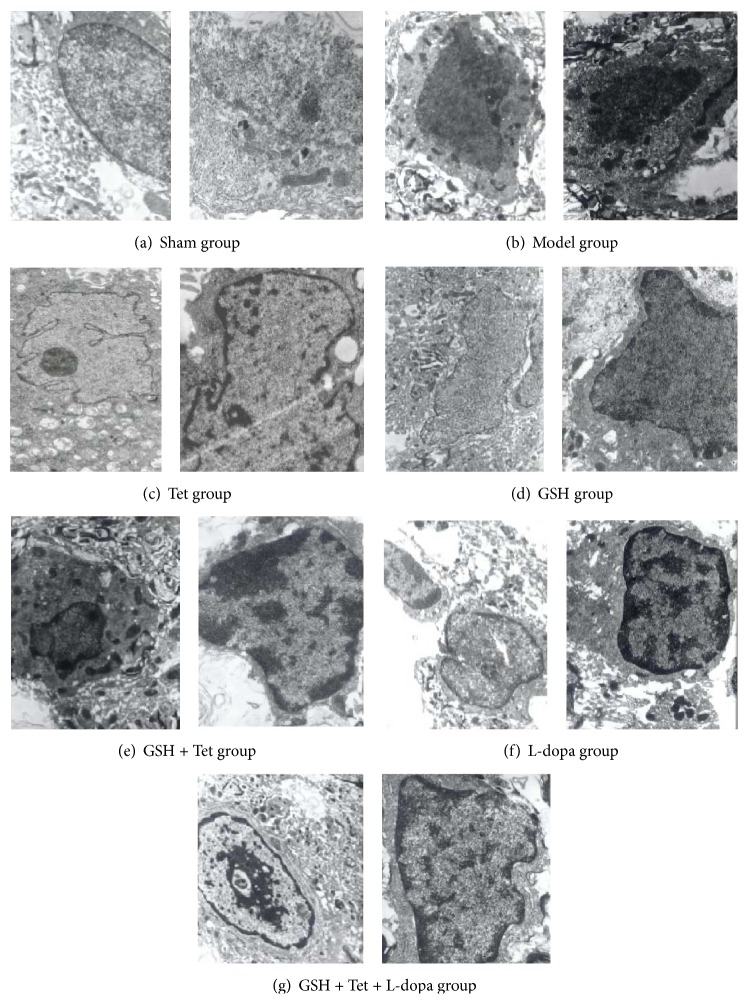
Transmission electron microscopy (TEM) images of apoptotic dopaminergic neuron in the substantia nigra pars compacta (SNc) of lesion sides. Images for all groups were taken using the same magnification (in one group, the left at 11,000x, the right at 15,000x). Dopaminergic neurons with normal cellular morphology were detected in the sham group. The model group showed characteristics of apoptosis in the early PD stage. The L-dopa group, however, displayed characteristics of apoptosis in the middle-late PD stage. In the Tet group and GSH groups, changes of apoptosis were less obvious compared with the L-dopa group. In the GSH + Tet and GSH + Tet + L-dopa group, several dopaminergic neurons were in preapoptosis stages.

To determine whether apoptosis was involved in the observed loss of dopaminergic neurons, we further examined the ultrastructure of dopaminergic neurons using TEM. As shown in Figure 6, dopaminergic neurons with normal cellular morphology were detected with only a few apoptotic cells in the sham group. However, in the model group, apoptotic morphological changes, such as reduced nuclear size, wrinkled nuclear envelope, nuclear chromatin condensation, and increased cytoplasmic density, were observed, characteristic of apoptosis in the early PD stage. In comparison with the model group, apoptotic cells in the L-dopa group displayed characteristics of apoptosis in the middle-late PD stage, including karyopyknosis, the appearance and margination of large and dense clumps of condensed chromatin, and swelling of organelles and karyorrhexis. In the Tet and GSH groups, changes such as karyopyknosis, condensation, and margination of chromatin and karyorrhexis were less obvious compared with the L-dopa group. Furthermore, several dopaminergic neurons in the GSH + Tet and GSH + Tet + L-dopa groups were in prechromatin condensation and margination stages, with a clearer subcellular structure.

## 4. Discussion

PD, characterized by a progressive and specific loss of dopaminergic neurons in the SNc, is the second most prevalent neurodegenerative disease after Alzheimer's disease (AD). Oxidative stress has been considered as an important pathophysiological process in PD [1], a condition which means that the sum of free radicals exceeds the antioxidants within a dopaminergic neuron. Endogenous DA, iron, neuromelanin, processes of normal DA metabolism, DA autoxidation, and enzymatic oxidation catalyzed by MAO of DA are all sources of free radicals in the SN [1, 9]. Under normal physiological conditions, free radicals are reduced or detoxicated by intrinsic antioxidant defenses. For PD patients, however, intrinsic antioxidant defenses within the SN are more fragile, and the most notable change is the decreased GSH levels. Postmortem studies of PD patients showed that GSH concentration in the SN decreased approximately by 40% compared with age-matched controls [2, 20].

GSH, a general thiol tripeptide, plays a vital role in maintaining the redox balance of neurons. Roughly speaking, by reducing free radicals, GSH protects dopaminergic neurons from oxidative damage [5]. To be specific, GSH not only acts directly in the detoxification of radicals but also serves as a substrate for various peroxidases [2]. Furthermore, GSH is a storage form and transfer way for cysteine, benefiting from maintaining redox potential [1, 5]. In several oxidative stress models, diminished GSH levels were shown to increase oxidative stress in whole cells as well as in mitochondrial fractions and to increase lipid peroxidation, intracellular calcium levels, and *γ*-glutamyl transpeptidase (*γ*GT) activity depletion [21, 22]. GSH depletion by buthionine sulfoximine (BSO), for example, potentiated the MPTP-induced TH-positive (TH+) neuronal death within the SN [23]. Consequently, researchers have thought that maintaining or restoring GSH levels in the SN would be a promising neuroprotective approach to delay the progression and limit the extent of dopaminergic neuronal loss in PD.

The overall GSH penetration into the CNS is limited, one reason why GSH has not been used to treat CNS diseases clinically [24]. In the present study, administration of GSH alone, as speculated, did not show significant effects on characteristics of PD rats. Coadministration of Tet with GSH, however, significantly decreased oxidative damage in the SN of PD rats, indicated by reduced levels of ROS, glutamate, aspartate, and MDA and increased GSH levels and CI activity in the GSH + Tet group as compared with the model and GSH groups. Accompanied with increased DA and HVA in the GSH + Tet group, decreased MAO-B and increased TH gene expression were observed in the GSH + Tet group, suggesting inhibited DA metabolism and enhanced DA synthesis. Furthermore, the GSH + Tet group showed increased TH-immunopositive neuronal cell survival and delayed apoptosis of dopaminergic neurons in the SN. Lastly, rotational behaviors of PD rats in the GSH + Tet group were significantly improved. Therefore, GSH + Tet showed several possible neuroprotective effects in the PD rat model. To exclude the possibility that Tet may influence the neurotransmitters, oxidative damage, and other parameters, we conducted a separate test in the Tet group, proving that Tet alone did not show any of the same effects as the GSH + Tet treatment on PD rats.

In the present study, we speculated that the increased neuroprotective effects of GSH when Tet was coadministered may be due to an inhibitory effect of Tet on P-gp, because Tet is a potent inhibitor of P-gp [13, 14, 25] and GSH is one substrate for P-gp [5]. P-gp acts as a drug efflux pump and serves as a primary obstacle for drug delivery to the brain. In several studies, anticancer drugs showed significantly increased effectiveness in brain tumors when P-gp inhibitors were coadministered, benefiting from the improved penetration of these drugs across the BBB [11]. Subsequently, in the present study the GSH brain penetration might be potentially improved due to an inhibitory effect of Tet on P-gp.

Tetrandrine, originating from traditional Chinese medicine of the root of* Stephania tetrandra* S. Moore, now is approved by State Drugs Administration of China as a new drug for the treatment of silicosis [26]. Tet is a potent, noncompetitive, and selective inhibitor of P-gp, showing a potent reversal effect on P-gp-mediated MDR in vitro and in vivo. Mechanism studies found that Tet could significantly inhibit the efflux and increase the uptake of P-gp substrates. Reportedly, Tet can significantly reduce P-gp gene expression [13, 14, 26, 27]. Tet toxicity, a major concern of researchers, may be linked to the dose, route, and rate of administration. Previous studies demonstrated that up to 150 mg/kg/day of Tet can be used via oral or intraperitoneal routes without causing significant side effects. Tet, as a relatively safe drug, can be given orally to humans at a dose of 200 mg or 300 mg/day for 3 months for the treatment of silicosis [26]. However, intravenous injection of tetrandrine (>3 mg/kg), particularly rapid intravenous injection of tetrandrine (>1 mg/kg/min), could induce significant toxic effects in vivo. The main toxicities are its negative inotropic and chronotropic effects on the heart, although they may also involve the liver, kidney, and lymphoid tissues [27]. In the present study, the intraperitoneal dose of Tet administered was only 15 mg/kg/day, a dose much less than 150 mg/kg/day. Despite all this, further toxicological studies about Tet are necessary.

The administration of L-dopa is a standard treatment for Parkinson's disease. Despite the marked benefits of L-dopa, its long-term use may cause adverse effects, especially motor fluctuations, as well as psychiatric symptoms. L-dopa-induced neurotoxicity mediated by generation of the reactive oxygen or nitrogen species has been reported in damaged neurons in many in vitro and in vivo studies. Furthermore, quinone generated from dopamine or L-dopa, which is closely linked to mitochondrial dysfunction, inflammation, and dysfunction of the ubiquitin-proteasome system, has received close attention as neuron-specific oxidative stress [28, 29]. The L-dopa quinone could exert cytotoxicity in or beside dopaminergic neurons by interacting with various bioactive molecules. In addition, L-dopa- and dopamine-induced cell death may result from induction of apoptosis, as evidenced by increases in caspase-3 activity [30].

In the present study, oxidative damage was aggravated slightly by L-dopa administration, represented by increased levels of ROS, MDA, glutamate, and aspartate and decreased levels of GSH and CI activity in the L-dopa group compared with the model group. The L-dopa group also showed increased MAO-B levels and decreased TH gene expression compared with the model group, indicating an elevated load of DA metabolism for dopaminergic neurons. Furthermore, L-dopa administration resulted in decreased TH-immunopositive dopaminergic neuronal cell survival. Finally, L-dopa administration may promote the apoptotic process of dopaminergic neurons. However, there is still some controversy regarding L-dopa treatment-induced pathogenicity in patients with Parkinson's disease. And some reports have shown that dopamine or L-dopa exerts either neuroprotective or even no effects on oxidative damage [17, 31, 32]. Despite all the uncertainty, the present study suggested that long-term administration of L-dopa might be involved in the process of oxidative stress in Parkinson's disease.

Previous studies demonstrated that formation of quinones and the consequent damage to dopaminergic neuronal cells could be successfully prevented by treatment with GSH in vitro and in vivo [33]. In the present study, reduced oxidative damage, elevated DA levels, increased TH-immunopositive dopaminergic neuronal cell survival, delayed apoptosis of dopaminergic neurons, and improved rotational behaviors of PD rats were observed in the GSH + Tet + L-dopa group compared with the L-dopa group. Thus, coadministration of GSH + Tet could counterbalance the possible oxidative neurotoxicity induced by L-dopa. Whether L-dopa is neurotoxic is controversial. Regardless, L-dopa remains the most efficacious treatment for motor symptoms of PD [34]. Therefore, coadministration of antioxidant GSH and the P-gp inhibitor Tet with L-dopa appears to be an ideal solution, not only for the etiology of PD but also due to the possible involvement of oxidative neurotoxicity in long-term L-dopa treatment.

The present study had several limitations. Based on previous as well as present studies, we speculated that enhanced antioxidant function of GSH may result from inhibitory effects of Tet on P-gp. However, the GSH concentration in the plasma and brain, P-gp expression in the PD brain, and changes of P-gp-mediated GSH efflux were not assessed in this study. Further studies should be conducted to assess the abovementioned limitations.

## 5. Conclusions

The current study demonstrated that tetrandrine allowed occurrence of the neuroprotective effect of glutathione probably due to inhibition of P-glycoprotein on 6-hydroxydopamine-lesioned rat models of Parkinson's disease, including the rats undergoing long-term L-dopa treatment. The mechanism may be related to tetrandrine inhibition on P-glycoprotein-mediated glutathione efflux in the blood-brain barrier. Although the potential use of P-glycoprotein inhibitors, such as tetrandrine, requires further research, the present study may offer a promising therapeutic alternative for restoring neuroprotective function of glutathione in Parkinson's disease. Additionally, to the best of our knowledge, this is the first study in which tetrandrine is involved in the treatment of Parkinson's disease.
